# Daily Eicosapentaenoic Acid Infusion in IUGR Fetal Lambs Reduced Systemic Inflammation, Increased Muscle ADRβ2 Content, and Improved Myoblast Function and Muscle Growth

**DOI:** 10.3390/metabo14060340

**Published:** 2024-06-18

**Authors:** Haley N. Beer, Taylor A. Lacey, Rachel L. Gibbs, Micah S. Most, Zena M. Hicks, Pablo C. Grijalva, Eileen S. Marks-Nelson, Ty B. Schmidt, Jessica L. Petersen, Dustin T. Yates

**Affiliations:** 1 Stress Physiology Laboratory, Department of Animal Science, University of Nebraska-Lincoln, Lincoln, NE 68583, USA; 2 Meat Science and Muscle Biology, Department of Animal Science, University of Nebraska-Lincoln, Lincoln, NE 68583, USA; ty.schmidt@unl.edu; 3 Animal Breeding and Genetics, Department of Animal Science, University of Nebraska-Lincoln, Lincoln, NE 68583, USA; jessica.petersen@unl.edu

**Keywords:** adaptive fetal programming, developmental origins of health and disease (DOHaD), low birthweight, maternofetal health, omega-3 polyunsaturated fatty acid (ω-3 PUFA), placental insufficiency, satellite cells, small for gestational age (SGA)

## Abstract

Intrauterine growth-restricted (IUGR) fetuses exhibit systemic inflammation that contributes to programmed deficits in myoblast function and muscle growth. Thus, we sought to determine if targeting fetal inflammation improves muscle growth outcomes. Heat stress-induced IUGR fetal lambs were infused with eicosapentaenoic acid (IUGR+EPA; *n* = 9) or saline (IUGR; *n* = 8) for 5 days during late gestation and compared to saline-infused controls (*n* = 11). Circulating eicosapentaenoic acid was 42% less (*p* < 0.05) for IUGR fetuses but was recovered in IUGR+EPA fetuses. The infusion did not improve placental function or fetal O_2_ but resolved the 67% greater (*p* < 0.05) circulating TNFα observed in IUGR fetuses. This improved myoblast function and muscle growth, as the 23% reduction (*p* < 0.05) in the ex vivo differentiation of IUGR myoblasts was resolved in IUGR+EPA myoblasts. *Semitendinosus*, *longissimus dorsi*, and *flexor digitorum superficialis* muscles were 24–39% lighter (*p* < 0.05) for IUGR but not for IUGR+EPA fetuses. Elevated (*p* < 0.05) IL6R and reduced (*p* < 0.05) β2 adrenoceptor content in IUGR muscle indicated enhanced inflammatory sensitivity and diminished β2 adrenergic sensitivity. Although IL6R remained elevated, β2 adrenoceptor deficits were resolved in IUGR+EPA muscle, demonstrating a unique underlying mechanism for muscle dysregulation. These findings show that fetal inflammation contributes to IUGR muscle growth deficits and thus may be an effective target for intervention.

## 1. Introduction

Asymmetrical intrauterine growth restriction (IUGR) of the fetus is, in large part, a product of disproportionally impaired skeletal muscle growth capacity [1,2]. The programming mechanisms that underlie slower fetal muscle growth occur in response to chronic O_2_ and nutrient deficits produced by placental insufficiency [3,4]. Although beneficial to the fetus, stress-induced adaptive programming of muscle results in lifelong deficits in lean muscle mass and metabolic efficiency after birth [3,4]. This markedly increases the risk for metabolic health disorders in IUGR-born individuals [5]. The same programming mechanisms cause poor growth efficiency and carcass composition in IUGR-born livestock [6,7]. Throughout late gestation, IUGR fetuses exhibit systemic inflammation and hypercatecholaminemia, which are instrumental in facilitating adaptations, including altered tissue sensitivity to both of these stress-regulating systems [8,9,10]. Near term, IUGR skeletal muscle exhibits reduced β2 adrenergic responsiveness and enhanced inflammatory tone that persists postnatally [11,12,13,14,15]. β2 adrenergic pathways increase nutrient uptake, protein synthesis, and metabolic rates in skeletal muscle [2,16,17,18,19,20] and thus are critical to efficient lean muscle growth [12,21,22,23]. Recent studies have confirmed the role of β2 adrenergic deficits in poor growth and metabolic function of IUGR muscle [24,25]. Conversely, heightened inflammatory activity disrupts the function of myoblasts (i.e., muscle stem cells) [26,27,28,29,30] and, in turn, impairs their ability to facilitate hypertrophic muscle growth [31,32,33]. In fact, experimental induction of sustained maternofetal inflammation alone produced an IUGR muscle phenotype similar to the one produced by heat stress-induced placental insufficiency [4,34]. Thus, we hypothesized that targeting enhanced inflammatory activity in the IUGR fetus would improve myoblast function, muscle growth, and body composition. Previous studies have shown that omega-3 polyunsaturated fatty acids (ω-3 PUFAs) have strong anti-inflammatory functions [35,36,37]. Moreover, studies in humans indicate that endogenous ω-3 PUFA is deficient in IUGR fetuses and offspring [38,39,40,41]. Therefore, we directly infused IUGR fetal lambs with the anti-inflammatory ω-3 PUFA, eicosapentaenoic acid, for 5 days and assessed the effects on inflammatory tone, myoblast function, and muscle growth.

## 2. Materials and Methods

### 2.1. Animals and Experimental Design

All procedures were reviewed and approved by the University of Nebraska-Lincoln’s Institutional Animal Care and Use Committee. Experiments were performed at the University of Nebraska-Lincoln, which is accredited by AAALAC International. After a 7-day acclimation period, Polypay ewes (2 to 4 years of age; 72.7 ± 0.4 kg; 2.5 to 3 body condition score) were timed-mated to a single Polypay male and heat-stressed to produce placental insufficiency-induced IUGR fetuses, as previously described [25,42]. Briefly, ewes carrying singleton or twin pregnancies were housed under ambient conditions of 40 °C and 35% relative humidity (86 temperature–humidity index per [43]) from the 40th to the 95th day of gestational age (dGA) and were then returned to thermoneutral conditions (25 °C, 35% relative humidity, 70 temperature–humidity index) for the duration of the study. Ewes carrying control fetuses were housed under static thermoneutral conditions and were pair-fed to the average daily intake of the heat-stressed ewes. Ewes were housed in adjacent individual pens with tenderfoot mesh flooring and were given approved environmental enrichment. Other aspects of standard husbandry practices, individual housing, and nutritional management were performed as previously described [4]. At 118 dGAs, partial cesarean surgeries were performed to place patent indwelling catheters into one fetal femoral artery and both femoral veins using the previously described hindlimb preparation procedure [4,44]. For twin pregnancies, only the fetus closest to the abdominal midline (i.e., incision site) was catheterized. From dGA 120 to 124, IUGR fetuses received daily IV infusions of the ω-3 PUFA eicosapentaenoic acid (0.25 mg/day; Cayman Chemical Co., Ann Arbor, MI, USA) over a 1 h period (i.e., IUGR+EPA; *n* = 9; 45% male, 56% twins) or a placebo infusion of saline carrier only (i.e., IUGR; *n* = 8; 55% male, 62% twins). Control fetuses (*n* = 11; 47% male, 63% twins) also received daily saline infusions. Simultaneous maternal venous (jugular venipuncture) and fetal arterial blood samples were collected 4 h after each daily infusion was completed to estimate glucose and O_2_ maternofetal gradients. Animals were euthanized via IV barbiturate overdose on dGA 125, representative placentomes were collected, and fetuses were necropsied. Weights were recorded for the whole fetus and the fetal hindlimb (dissected as previously described [45]), *semitendinosus*, *soleus*, *longissimus dorsi*, and *flexor digitorum superficialis* muscles, heart, lungs, liver, kidneys, and brain.

### 2.2. Blood Sample Analyses

Blood samples were collected daily from dGA 120 to 124 and analyzed for gas, metabolite, and cellular components, as previously described [24,25]. Briefly, daily fetal arterial and maternal venous whole blood samples were simultaneously collected into heparinized syringes and analyzed with an ABL90 FLEX (Radiometer, Brea, CA, USA) for glucose concentrations and partial pressures of O_2_ (pO_2_). Daily fetal blood samples were also collected into EDTA syringes and analyzed with a HemaTrue veterinary hematology analyzer (Heska Corp., Loveland, CO, USA) for concentrations of total white blood cells, granulocytes, monocytes, lymphocytes, platelets, and red blood cells, as well as hematocrit, hemoglobin concentration, mean corpuscular volume, mean corpuscular hemoglobin concentration, mean packed cell volume, and red blood cell distribution width. Blood plasma was separated from EDTA-treated whole blood by centrifugation (14,000× *g*, 2 min) and stored at −80 °C. Commercial ELISA kits were used to determine plasma concentrations of TNFα (Wuhan Biotech, Wuhan, China) and eicosapentaenoic acid (MyBioSource, Inc., San Diego, CA, USA) in duplicate, as previously described [25,46]. Coefficients of variance (inter- and intra-assay) were less than 15% for both ELISAs.

### 2.3. Tissue Sample Analyses

#### 2.3.1. Histology and Immunohistochemistry

Muscle fiber size and myoblast profiles were determined in fetal *semitendinosus* muscles using immunohistochemistry, as previously described [24,42]. Sections of the muscle were fixed in a paraformaldehyde solution (4% in phosphate-buffered saline; PBS; MilliporeSigma, St. Louis, MO, USA), embedded in cassettes using OCT compound (Scigen Scientific, Gardena, CA, USA), and stored frozen at −80 °C. Cross-sections (8 μm) were taken a minimum of 100 μm apart with a CryoStar NX50 cryostat (Richard-Allen Scientific Co., Kalamazoo, MI, USA) and placed on Fisher Superfrost Plus glass microscope slides. Mouse monoclonal IgG1 antibody raised against desmin (1:100; DE-U-10; GeneTex, Irvine, CA, USA) was used to stain muscle fibers in order to determine the average cross-sectional fiber area. Mouse monoclonal IgG1 antibody raised against pax7 (1:10; PAX7; DSHB, Iowa City, IA, USA), mouse monoclonal IgG1 antibody raised against myogenin (1:10; F5D; DSHB), and rabbit recombinant monoclonal IgG antibody raised against proliferating cell nuclear antigen (PCNA; 1:10; CPTC-PCNA-1; DSHB) were used to estimate myoblast population dynamics. Immunocomplexes were detected with Alexa Fluor 488, 555, or 594 secondary antibodies (1:1000; Invitrogen; Carlsbad, CA, USA). Sections were counterstained with the pan nuclei indicator DAPI (Fluoromount-G; Southern Biotech, Birmingham, AL, USA). Images were visualized on an Olympus IX73 microscope (Shinjuku, Tokyo, Japan) and were captured digitally with an Olympus DP80 camera. Analyses were performed on de-identified images using Olympus cellSense Dimension 1.13 software. The average *semitendinosus* muscle fiber area was estimated for each fetus from a minimum of 250 desmin^+^ fibers, as previously described [24,47]. Total myoblasts were estimated from the percentage of total nuclei (i.e., DAPI^+^) that were also pax7^+^. Proliferating myoblasts were estimated from the percentage of myoblasts (i.e., pax7^+^) that were also PCNA^+^. Differentiated myoblasts were estimated from the percentage of total nuclei (i.e., DAPI^+^) that were also myogenin^+^. Myoblast profiles were determined from a minimum of 1500 total nuclei, as previously described [24,47]. The three intact placentomes closest to the uterine bifurcation (i.e., uteroplacental incision site) were collected at necropsy and prepared for staining as previously described [48,49], with some modifications. Briefly, placentomes were halved longitudinally, fixed in 4% paraformaldehyde, and embedded in an OCT compound. Cross-sections (10 µm) were mounted on Fisher Superfrost Plus glass slides at −20 °C, brought to room temperature, and dried. For lipid staining, placentome sections were washed with 60% isopropanol, incubated with a working solution of Oil Red O (MilliporeSigma) for 15 min, and then washed again with 60% isopropanol. Sections were then rinsed with de-ionized H_2_O, cover-slipped, and stored at 4 °C. Sections were also stained for collagen using the commercial Gomori’s Trichrome Staining Kit (Richard-Allan Scientific, San Diego, CA, USA) following the manufacturer’s protocol. Lipid droplets and collagen were visualized with an Olympus IX73 microscope, and images were captured with an Olympus DP80 camera. ImageJ software (ImageJ 1.x, National Institutes of Health, Bethesda, MD, USA) was utilized to quantify lipid droplet populations and collagen^+^ area. Lipid droplet metrics were estimated from an average of 5000 droplets assessed across nine non-overlapping fields of view. The collagen^+^ area was estimated from six non-overlapping fields of view that were 10 mm^2^ in size.

#### 2.3.2. Protein Immunoblots

Total protein isolated from snap-frozen *semitendinosus* was used to determine the content of β2 adrenoceptor and interleukin-6 receptor (IL6R), as previously described [24,25]. Briefly, muscle samples were homogenized by sonication in lower salt extraction buffer containing 20 mM TRIS, 80 mM NaCl, 2.7 mM KCl, 1 mM MgCl_2_, 1 mM EDTA, 0.1% SDS, 1% Triton-X 100, 10% glycerol, 2.5% protease, and 2.5% phosphatase inhibitor, and total protein was isolated by 5 min centrifugation at 14,000× *g*. Supernatant protein concentrations were determined using Piece BCA Assay (Thermo Fisher, Waltham, MA, USA), and 50-μg aliquots were mixed with 4x Laemmli buffer (Bio-Rad Laboratories, Hercules, CA, USA). Samples were heated for 5 min at 95 °C, cooled to room temperature, separated via SDS-PAGE, and then transferred to Bio-Rad poly-vinylidene fluoride low-fluorescent membranes. These membranes were incubated in Bio-Rad EveryBlot Blocking Buffer, washed with TBS-T, and incubated with rabbit anti-serum raised against β2 adrenoceptor (1:1000, Cohesion Biosciences, London, UK) or against IL6R (1:1000, EPR24322-143; Abcam; Cambridge, MA, USA) overnight at 4 °C. Membranes were then washed with TBS-T, incubated for 1 h with goat anti-rabbit IR800 IgG secondary anti-serum (LI-COR Biosciences, Lincoln, NE, USA), imaged with the LI-COR Odyssey Infrared system, and quantified with LI-COR Image Studio Lite 5.2.

### 2.4. Ex Vivo Myoblast Function

#### 2.4.1. Primary Myoblast Isolation

Fetal myoblasts were isolated from hindlimb muscle at necropsy as previously described [29,50]. Briefly, the muscle was washed with cold PBS + antibiotic-antimycotic solution (1%; AbAm; Gibco, Grand Island, NY, USA) + gentamicin (0.5%; Gibco), finely minced, and digested for 1 h at 37 °C in PBS + protease type XIV from *Streptococcus griseus* (1.25 mg/mL; MilliporeSigma). Digested muscle was serial-centrifuged for 10, 8, and 1 min at 500× *g*. The supernatant was centrifuged for 5 min at 1500× *g* to separate isolated myoblasts, which were re-suspended and expanded in complete growth media (i.e., Dulbecco’s Modified Eagle’s Media (DMEM; Gibco) + 20% fetal bovine serum (FBS, Atlas Biologicals, Ft. Collins, CO, USA) + 1% AbAm, + 0.5% gentamicin) on fibronectin-coated (10 µg/mL; MilliporeSigma) tissue culture plates. Myoblast isolates were cryopreserved in complete growth media + 10% dimethyl sulfoxide (MilliporeSigma) over liquid nitrogen. The purity of each myoblast isolate was determined by staining subsamples of cells of pax7.

#### 2.4.2. Myoblast Proliferation

Myoblasts (4000 cells/well) were grown for 72 h in complete growth media on 6-well fibronectin-coated plates and then incubated for 24 h in complete growth media + 0 or 5 mU/mL insulin (Humulin R; Lilly, Indianapolis, IN, USA). For the final 2 h, myoblasts were pulse-labeled with 10 nM EdU (Thermo Fisher, Waltham, MA, USA). After brief cooling, myoblasts were lifted with Accutase and fixed in suspension with 4% paraformaldehyde. Proliferation rates were estimated from EdU^+^ myoblasts, which were identified with the ClickIT EdU Alexa Fluor 555 Cell Proliferation Assay (Life Technologies, Carlsbad, CA, USA), as previously described [29]. Percentages of EdU^+^ myoblasts were determined using flow cytometry with an ORFLO zEPI (Ketchum, ID, USA).

#### 2.4.3. Myoblast Differentiation

Myoblasts (20,000 cells/well) were differentiated by 96 h incubation in differentiation media (DMEM + 2% FBS + 1% AbAm + 0.5% gentamicin) containing 0 or 5 mU/mL insulin. After cooling on ice for 2 min, cells were lifted from plates and fixed in 4% paraformaldehyde. Myoblasts were then stained in suspension for myogenin (1:50; F5B; BD Pharmingen, Franklin Lakes, NJ, USA) and secondary affinity-purified anti-mouse IgG PE-Conjugate antibody (1:250; Cell Signaling Technology, Danvers, MA, USA), as previously described [29]. Percentages of myogenin^+^ cells were determined with flow cytometry.

### 2.5. Statistical Analysis

Histology, immunoblot, and biometric data were analyzed with ANOVA using the mixed procedure of SAS 9.4 (SAS Institute, Cary, NC, USA) to determine the fixed effects of the experimental group (control, IUGR, IUGR+EPA), sex (male, female), and birth number (singleton, twin). Fetus was used as a random effect. Interactions among these main effects were not included due to limited power. Mean separation for the experimental group effect was performed via the Fisher LSD test. Components of daily fetal blood samples and maternofetal blood gradients were analyzed via the mixed procedure of SAS with repeated measures for the fixed effects of the experimental group, fetal age, and the interaction, as well as sex and birth number. The fetus was the individual experimental unit. Significant differences were indicated by *p*-values of less than 0.05 and tendencies by *p*-values of less than 0.10. Data are presented as means ± standard errors of the mean.

## 3. Results

### 3.1. Placental Function Indicators

#### 3.1.1. Histology

**Figure 1:**
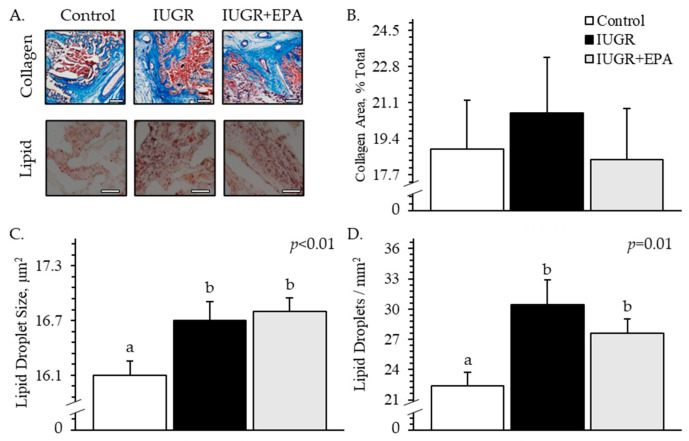
Lipid accumulation and fibrotic area in placentomes from IUGR fetal lambs administered daily with eicosapentaenoic acid. Representative images for Trichrome (top row, scale bar = 400 μm) and Oil Red O (bottom row, scale bar = 50 μm) staining are shown in frame (**A**). Staining was performed in control (*n* = 11), IUGR (*n* = 8), and IUGR+EPA fetuses (*n* = 9). Data are presented for relative collagen area (**B**), average lipid droplet size (**C**), and lipid droplet density (**D**). Effects of the experimental group were evaluated and are noted where significant (*p* < 0.05). ^a,b^ Means with different superscripts differ (*p* < 0.05).

Representative micrographs of collagen and lipid staining are shown in Figure 1A. The percentage of collagen^+^ placentome area did not differ between the experimental groups (Figure 1B). Average lipid droplet size was greater (*p* < 0.05) for placentomes from IUGR and IUGR+EPA pregnancies than from controls (Figure 1C). Lipid droplet density was greater (*p* < 0.05) for placentomes from IUGR and IUGR+EPA pregnancies than from controls (Figure 1D). No differences in placentome collagen or lipid droplet size were observed between males and females or between singletons and twins. Lipid droplet density was lower (*p* < 0.05) for placentomes from twin pregnancies than from singletons.

#### 3.1.2. Blood Glucose and O_2_

**Figure 2:**
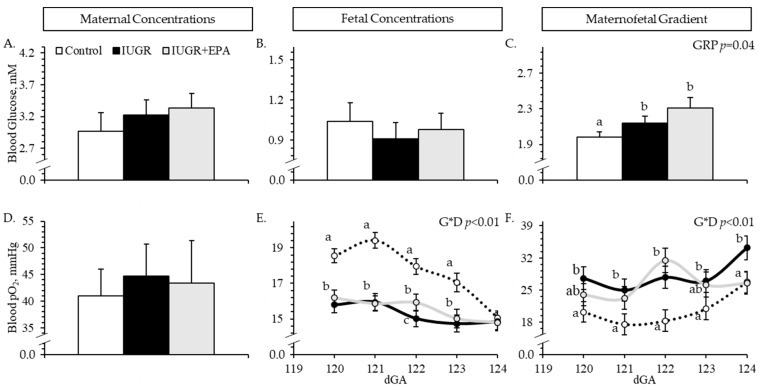
Placental insufficiency indicators in IUGR fetal lambs administered daily with eicosapentaenoic acid. Daily whole blood samples were collected from control (*n* = 11), IUGR (*n* = 8), and IUGR+EPA fetuses (*n* = 9) simultaneously with maternal blood samples. On the top row, data are presented for maternal glucose (**A**), fetal glucose (**B**), and maternofetal glucose gradients (**C**). On the bottom row, data are presented for maternal pO_2_ (**D**), fetal pO_2_ (**E**), and maternofetal pO_2_ gradient (**F**), Effects of the experimental group (GRP), day of gestation, and group x day interaction (G*D) were evaluated and are noted where significant (*p* < 0.05). ^a–c^ Means with different superscripts differ (*p* < 0.05).

Experimental group × dGA interactions were observed (*p* < 0.05) for fetal blood pO_2_ and maternofetal pO_2_ gradient but not for any other gradient variables. Maternal and fetal blood glucose concentrations did not differ between experimental groups (Figure 2A and Figure 2B, respectively), but the maternofetal glucose gradient was greater (*p* < 0.05) for IUGR and IUGR+EPA pregnancies than for controls (Figure 2C). Maternal blood pO_2_ did not differ between groups (Figure 2D). Fetal blood pO_2_ was lower (*p* < 0.05) for IUGR and IUGR+EPA fetuses than for controls on all days except dGA 124, where it did not differ between groups (Figure 2E). Fetal blood pO_2_ was also intermediate for IUGR+EPA fetuses (i.e., between controls and IUGR fetuses) on dGA 122. Maternofetal pO_2_ gradient was lower (*p* < 0.05) for IUGR pregnancies than for controls on all days and was lower (*p* < 0.05) for IUGR+EPA pregnancies than controls for dGAs 121 and 122 but not dGAs 120, 123, or 124 (Figure 2F). No parameters differed between males and females, but maternofetal glucose gradients were greater (*p* < 0.05), and fetal pO_2_ was lower (*p* < 0.05) for twins than singletons.

### 3.2. Fetal Hematology

#### 3.2.1. Circulating Leukocytes

**Figure 3:**
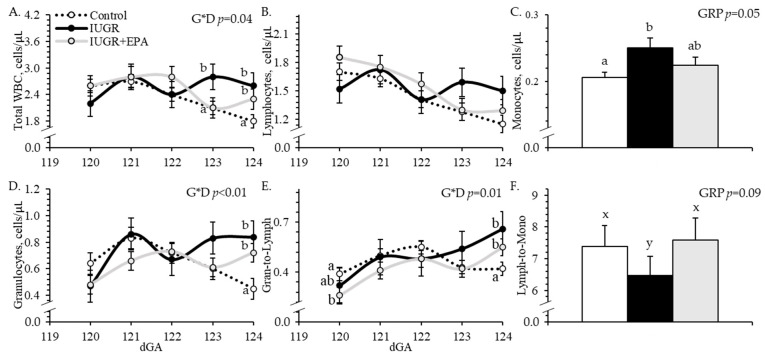
Circulating leukocytes in IUGR fetal lambs administered daily with eicosapentaenoic acid. Complete blood counts were performed on daily whole blood samples collected from control (*n* = 11), IUGR (*n* = 8), and IUGR+EPA fetuses (*n* = 9). Data are presented for circulating concentrations of total white blood cells (**A**), lymphocytes (**B**), monocytes (**C**), and granulocytes (**D**), as well as granulocyte-to-lymphocyte (**E**) and lymphocyte-to-monocyte ratios (**F**). Effects of the experimental group (GRP), day of gestation, and group x day interaction (G*D) were evaluated and are noted where significant (*p* < 0.05). ^a,b^ Means with different superscripts differ (*p* < 0.05). ^x,y^ Means with different superscripts tend to differ (*p* < 0.10).

Experimental group × dGA interactions were observed (*p* < 0.05) for circulating total white blood cells, granulocytes, and granulocyte-to-lymphocyte ratios but not for any other leukocyte parameters. Circulating white blood cell concentrations did not differ between groups on dGA 120, 121, and 122, were greater (*p* < 0.05) for IUGR but not for IUGR+EPA fetuses than for controls on dGA 123 and were greater (*p* < 0.05) for IUGR and IUGR+EPA fetuses than for controls on dGA 124 (Figure 3A). Circulating lymphocyte concentrations did not differ between groups for any day (Figure 3B). Circulating monocyte concentrations were greater (*p* < 0.05) for IUGR but not for IUGR+EPA fetuses than for controls, regardless of day (Figure 3C). Circulating granulocyte concentrations did not differ between groups on any day except dGA 124, where they were greater (*p* < 0.05) for IUGR and IUGR+EPA fetuses than for controls (Figure 3D). Granulocyte-to-lymphocyte ratios were lower (*p* < 0.05) for IUGR+EPA but not IUGR fetuses than for controls on dGA 120, did not differ between groups on dGAs 121, 122, and 123, and were greater (*p* < 0.05) for IUGR and IUGR+EPA fetuses than for controls on dGA 124 (Figure 3E). Lymphocyte-to-monocyte ratios tended to be lower (*p* < 0.10) for IUGR but not for IUGR+EPA fetuses than for controls (Figure 3F). No differences in leukocyte concentrations were observed between males and females or between singletons and twins.

#### 3.2.2. Hematological Parameters

No experimental group × dGA interactions were observed for any hematological parameters, which are presented in Supplemental Figure S1. Fetal hematocrit, red blood cell distribution width, hemoglobin, mean corpuscular hemoglobin concentration, red blood cell concentrations, and mean packed volume did not differ between experimental groups. Mean corpuscular volume was lower (*p* < 0.05) for IUGR fetuses and greater (*p* < 0.05) for IUGR+EPA fetuses than for controls, regardless of day. Circulating platelet concentrations were greater (*p* < 0.05) for IUGR and IUGR+EPA fetuses than for controls, regardless of day. No differences in hematology parameters were observed between males and females, but mean corpuscular volume was greater (*p* < 0.05) and mean corpuscular hemoglobin concentrations were smaller (*p* < 0.05) for twins than for singletons.

### 3.3. Circulating Eicosapentaenoic Acid and TNFα

**Figure 4:**
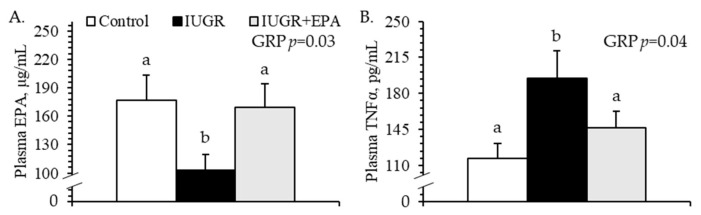
Systemic inflammation in IUGR fetal lambs administered daily with eicosapentaenoic acid. Plasma was isolated from daily blood samples collected from control (*n* = 11), IUGR (*n* = 8), and IUGR+EPA fetuses (*n* = 9). Data are presented for fetal plasma eicosapentaenoic acid (**A**) and TNFα (**B**) concentrations. Effects of the experimental group (GRP), day of gestation, and group × day interaction were evaluated and are noted where significant (*p* < 0.05). ^a,b^ Means with different superscripts differ (*p* < 0.05).

No experimental group × dGA interactions were observed for circulating eicosapentaenoic acid or TNFα. Circulating eicosapentaenoic acid concentrations were smaller (*p* < 0.05) for IUGR but not for IUGR+EPA fetuses than for controls, regardless of day (Figure 4A). Conversely, circulating TNFα concentrations were greater (*p* < 0.05) for IUGR but not for IUGR+EPA fetuses than for controls, regardless of day (Figure 4B). No differences in eicosapentaenoic acid or TNFα were observed between singletons and twins, but circulating TNFα was lower (*p* < 0.05) for male fetuses than for female fetuses.

### 3.4. Fetal Biometrics

At necropsy, body weights were lighter (*p* < 0.05) for IUGR but not IUGR+EPA fetuses than for controls (Table 1). Fetal hindlimb weights were lower (*p* < 0.05) for IUGR fetuses than for controls and IUGR+EPA fetuses. *Semitendinosus*, *longissimus dorsi*, and *flexor digitorum superficialis* muscles were lighter (*p* < 0.05) for IUGR but not for IUGR+EPA fetuses than for controls. *Soleus* muscles tended to be lighter (*p* < 0.10) for IUGR fetuses than for controls and tended to be intermediate for IUGR+EPA fetuses. Hearts and lungs were lighter (*p* < 0.05) for IUGR and IUGR+EPA fetuses than for controls. Liver weights did not differ between groups. Kidneys and brains were lighter (*p* < 0.05) for IUGR but not IUGR+EPA fetuses than for controls. Heart/bodyweight, lungs/bodyweight, liver/bodyweight, and kidneys/bodyweight did not differ between groups. Brain/bodyweight was greater (*p* < 0.05) for IUGR fetuses than for controls or IUGR+EPA fetuses. Fetal *soleus* and *flexor digitorum superficialis* muscles were heavier (*p* < 0.05) and lung/bodyweight and brain/bodyweight were lower (*p* < 0.05) for males than for females. No differences in biometrics were observed between singletons and twins.

### 3.5. Muscle GROWTH and Regulation

#### 3.5.1. Receptor Content

**Figure 5:**
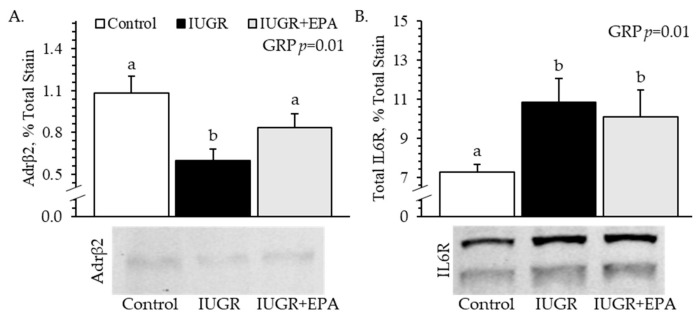
Skeletal muscle hormone receptor content for IUGR fetal lambs administered daily with eicosapentaenoic acid. Total protein was isolated from *semitendinosus* muscle samples collected from control (*n* = 11), IUGR (*n* = 8), and IUGR+EPA fetuses (*n* = 9). Data are presented for protein immunoblot analysis of muscle β2 adrenoceptor (**A**) and IL-6 receptor (**B**) content. Effects of the experimental group (GRP) were evaluated and are noted where significant (*p* < 0.05). ^a,b^ Means with different superscripts differ (*p* < 0.05).

β2 adrenoceptor protein content was lower (*p* < 0.05) for *semitendinosus* muscles from IUGR fetuses than from IUGR+EPA fetuses and controls (Figure 5A). Total IL6R protein content was higher (*p* < 0.05) for *semitendinosus* muscles from IUGR and IUGR+EPA fetuses than from controls (Figure 5B). The protein content of the IL6R isoform in the larger band was also greater (*p* < 0.05) for *semitendinosus* muscles IUGR and IUGR+EPA fetuses than from controls, but the protein content of the IL6R isoform in the smaller band did not differ between groups. The protein content of the larger isoform, smaller isoform, and total IL6R was higher (*p* < 0.05) in *semitendinosus* muscle from males than from females. No differences in receptors were observed between singletons and twins.

#### 3.5.2. Myoblast Profiles and Fiber Size

**Figure 6:**
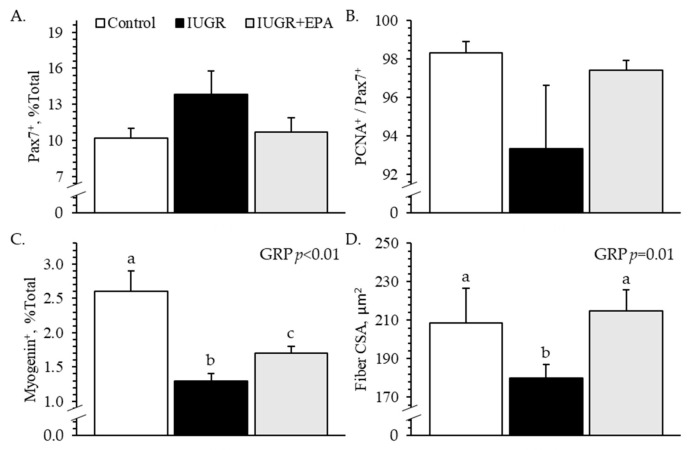
Myoblast profiles in skeletal muscle from IUGR fetal lambs administered daily with eicosapentaenoic acid. Immunohistochemistry was performed on fixed *semitendinosus* muscle cross-sectional samples collected from control (*n* = 11), IUGR (*n* = 8), and IUGR+EPA fetuses (*n* = 9). Data are presented for total myoblasts (**A**), proliferating myoblasts (**B**), differentiated myoblasts (**C**), and average cross-sectional muscle fiber area (**D**). Effects of the experimental group (GRP) were evaluated and are noted where significant (*p* < 0.05). ^a–c^ Means with different superscripts differ (*p* < 0.05). Representative staining images are included in the Supplemental Materials.

Representative micrographs for immunohistological staining of *semitendinosus* muscles are presented in Supplemental Figure S2. The percentage of pax7^+^ nuclei (i.e., total myoblasts) in cross-sections of the fetal *semitendinosus* muscle did not differ between experimental groups (Figure 6A). Likewise, the percentage of pax7^+^/PCNA^+^ nuclei (i.e., proliferating myoblasts) in cross-sections of the fetal *semitendinosus* muscle did not differ between groups (Figure 6B). However, the percentage of myogenin^+^ nuclei (i.e., differentiated myoblasts) was smaller (*p* < 0.05) for *semitendinosus* muscles from IUGR fetuses than from controls and was intermediate for IUGR+EPA fetuses (Figure 6C). The average cross-sectional area of *semitendinosus* muscle fibers was smaller (*p* < 0.05) for IUGR fetuses than for controls or IUGR+EPA fetuses (Figure 6D). No differences in myoblast profiles were observed between males and females, but myogenin^+^ nuclei were fewer (*p* < 0.05) and fiber size was larger (*p* < 0.05) for twins than for singletons.

#### 3.5.3. Ex Vivo Myoblast Function

**Figure 7:**
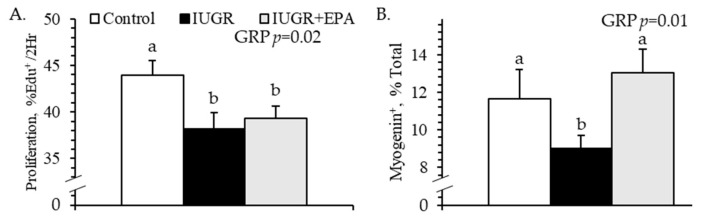
Ex vivo myoblast function for IUGR fetal lambs administered daily with eicosapentaenoic acid. Primary myoblasts were isolated from the hindlimb muscles of control (*n* = 11), IUGR (*n* = 8), and IUGR+EPA fetuses (*n* = 9) and studied in culture. Data are presented for proliferation rates (**A**) during a 2 h EdU pulse and for differentiation rates (**B**) following a 4-day induction of differentiation. Effects of the experimental group (GRP), incubation media, and group × media interaction were evaluated and are noted where significant (*p* < 0.05). ^a,b^ Means with different superscripts differ (*p* < 0.05).

Pax7^+^ staining indicated that the average purity of primary fetal myoblast isolates was 92.4%. Proliferation rates during the 2 h EdU pulse were lower (*p* < 0.05) for myoblasts isolated from IUGR and IUGR+EPA fetuses than from controls, regardless of media insulin concentration (Figure 7A). Differentiation rates (i.e., myogenin^+^ nuclei after ex vivo differentiation) were lower (*p* < 0.05) for myoblasts isolated from IUGR fetuses than those isolated from controls or IUGR+EPA fetuses (Figure 7B). No differences were observed in ex vivo myoblast function between males and females or between singletons and twins.

## 4. Discussion

In this study, we found that targeting systemic inflammation in IUGR fetal sheep with a daily infusion of the ω-3 polyunsaturated fatty acid eicosapentaenoic acid recovered myoblast differentiation capacity and improved muscle hypertrophy. These improvements occurred despite no meaningful amelioration of placental insufficiency or the associated fetal hypoxemia. Stress conditions during pregnancy can increase lipid accumulation, inflammation, and fibrosis in placental tissues [51,52,53], which in turn diminishes placental function [54,55]. Maternal hyperthermia in the present study resulted in larger and more abundant lipid droplets within placentome tissues. Although the fibrotic area was not increased at this stage of pregnancy, the placental transfer of O_2_ to the fetus was nevertheless diminished by about 37%. Physiological hypoxia stimulates strong inflammatory responses from leukocytes [56,57,58]. This was characterized in our IUGR fetuses by persistently greater numbers of cytokine-producing monocytes in the bloodstream and an almost 70% elevation in circulating TNFα concentrations. Fetal hypoxemia is resolved by birth, and circulating monocyte numbers consequently return to normal in IUGR offspring [24]. However, we recently found that blood cytokine concentrations remained elevated well after birth [24,25], which is consistent with the programming of a more inflammatory phenotype in IUGR monocytes [59]. In the present study, systemic inflammation in IUGR fetuses also coincided with a 42% reduction in circulating concentrations of the anti-inflammatory ω-3 PUFA eicosapentaenoic acid, which was presumably a product of stress-impaired Δ5 and Δ6 desaturase activity, as previously observed [60,61,62,63]. The loss of endogenous eicosapentaenoic acid may also help to explain enhanced inflammatory sensitivity in IUGR muscle, illustrated by greater IL6R protein content in this study and by greater TNFR1 and other canonical pathway components in previous studies [29,34,64]. As fetal infusion of eicosapentaenoic acid brought circulating concentrations back to normal, monocyte and TNFα concentrations returned to normal in kind. In contrast, concentrations of other white blood cells remained elevated, indicating that the anti-inflammatory effects were primarily directed at monocytes. Cell culture studies show that ω-3 PUFA moderates monocytic activity by inhibiting canonical NFκB, p38 MAPK, and TLR4 pathways [65,66,67], which in turn reduce cytokine production and secretion [68]. Similar outcomes were reported when ω-3 PUFA was used to treat chronic inflammatory conditions in humans, as monocytic populations and activity were suppressed with little or no effect on other leukocytes [69,70,71].

Disrupting systemic inflammation in IUGR fetuses improved indicators of body composition and muscle growth. Hallmark asymmetry of the IUGR fetus arises from the preferential sparing of vital brain and heart growth at the expense of muscle and adipose accretion [72,73]. These programmed patterns of disproportional tissue growth persist in IUGR-born offspring [13,72,74]. Although reduced fat deposition is resolved postnatally, reduced muscle mass remains a lifelong deficiency [24,75,76]. In our IUGR fetuses, asymmetric body composition was clearly associated with the disproportionate restriction of muscle growth. To illustrate, IUGR fetal body weights were reduced by 21%, but individual muscle weights were reduced by 24% to 39%. Moreover, fetal hindlimbs, which are up to two-thirds skeletal muscle [45], were 26% lighter than normal. By comparison, IUGR fetal brain and heart weights were reduced by only 10% and 16%, respectively. Greater brain-to-bodyweight ratios in IUGR fetuses were perhaps the most indicative of hallmark brain sparing. Not surprisingly, the resolution of asymmetric body composition following eicosapentaenoic acid infusion was associated with an approximate 50% recovery in weights for individual muscles and hindlimbs. Clinical studies have shown that long-term ω-3 PUFA supplementation can increase muscle mass in healthy individuals without increasing adiposity [77,78] and can slow or even prevent muscle atrophy under pathological conditions [79,80]. Similarly, ω-3 PUFA supplementation to meat animals increased carcass weight, lean muscle yield, and size of individual meat cuts without increasing carcass fat [81,82]. Supplementing ω-3 PUFA-rich fish oil also preserved muscle growth rates in lambs during prolonged heat stress by circumventing systemic inflammation [83,84].

Improved muscle growth in eicosapentaenoic acid-infused IUGR fetuses was facilitated by the recovery of myoblast function. Because of their role in muscle fiber hypertrophy, the intrinsic functional impairment of IUGR myoblasts is rate-limiting for muscle growth [29,50]. Ex vivo assessments in the present study revealed a modest deficit in the proliferative capacity of IUGR fetal myoblasts that was not apparent when myoblast populations of the *semitendinosus* muscle were profiled by staining. However, the differentiation capacity of IUGR myoblasts was substantially impaired, as illustrated by 23% fewer myogenin-positive cells following ex vivo induction and 51% fewer myogenin-positive nuclei in the IUGR *semitendinosus*. Previous cell culture experiments have demonstrated that TNFα and other inflammatory mediators are particularly strong inhibitors of myoblast differentiation and fusion [85,86,87,88,89,90]. This suppressive effect occurs largely via canonical pathways, as the inhibition of individual membrane receptors and downstream second messengers like TRAF6 and NFκB diminished much of the inhibitory effect [28,90,91,92]. A recent study from our lab found that IUGR fetal myoblasts develop enhanced sensitivity to inflammatory cytokines [29], which is also evident in their muscle tissues [34,64]. Additional studies demonstrated that fetal inflammation alone is sufficient to suppress hypertrophic muscle growth before and after birth [4,34]. Consequently, targeting systemic fetal inflammation in the present study markedly improved myoblast differentiation capacity, which in turn contributed to the recovery of muscle fiber size.

Improved IUGR fetal muscle growth with eicosapentaenoic acid was not facilitated by a resolution in elevated muscle IL6R content, which remained uncorrected following the infusion regimen. This was somewhat unexpected, as previous literature led us to postulate that sustained the alleviation of systemic inflammation might circumvent enhanced inflammatory pathways. Specifically, canonical IL6R pathways are known to suppress muscle growth [93,94], and IL6R expression was increased in the hindlimb muscles of fetal rats following chronic intrauterine inflammation, resulting in a smaller muscle size [95]. Assessments of ω-3 PUFA exposure on IL6R-mediated pathways in skeletal muscle are limited in the current literature. However, incubation of epithelial cells with ω-3 PUFA or their bioactive metabolites reduced gene and protein expression for IL6R as well as several downstream signaling components [96,97]. Even though skeletal muscle IL6R content remained elevated following eicosapentaenoic acid infusion in our IUGR fetuses, it is possible that pathways were disrupted further downstream. For example, the anti-inflammatory effects of ω-3 PUFA in myoblast cell lines were at least partially facilitated by the upregulation of PPARγ, which is a disruptor of NFκB activity [98,99]. Thus, a robust assessment of downstream changes in addition to cytokine receptors is a warranted component of future IUGR intervention studies. In an additional unexpected observation, the deficit in skeletal muscle β2 adrenoceptor content observed in our IUGR fetuses was resolved with eicosapentaenoic acid infusion. This represents a key potential mechanistic explanation for the programmed dysregulation of IUGR muscle growth, as well as a target for its resolution. Under normal conditions, β2 adrenergic pathways stimulate muscle protein synthesis, myoblast activity, and hypertrophic growth [100,101,102,103,104]. In IUGR-born offspring, however, skeletal muscle β2 adrenoceptor content and activity are reduced [12,13,24]. The pharmaceutical stimulation of β2 adrenergic activity in these IUGR-born offspring helped improve muscle growth and metabolic function but did not correct the β2 adrenoceptor deficit [24]. Regulation by inflammatory and β2 adrenergic systems can be antagonistic, and β2 adrenergic stimulation has been shown to dampen canonical inflammatory signaling pathways in skeletal and smooth muscle [105,106,107]. Additionally, administering β2 agonists suppressed elevated circulating cytokines in IUGR-born lambs [25] and heat-stressed livestock [108,109]. Inversely, experimentally heightened inflammation in rodent models reduced β2 adrenergic tone in skeletal muscle [110], smooth muscle [111,112,113,114], cardiomyocytes [115,116], and alveolar leukocytes [117]. Moreover, the incubation of primary brain cells with ω-3 PUFA increased β2 adrenoceptor content and intracellular cAMP concentrations [108,109,118]. Chronic hypercatecholaminemia and systemic inflammation are both hallmark conditions of the IUGR fetus [2,64], and we previously presumed that reduced skeletal muscle β2 adrenoceptor was solely a product of the former [119,120]. However, our present findings indicate that systemic inflammation is, in fact, a key contributor to the loss of β2 adrenergic tone. Therefore, therapeutic intervention with anti-inflammatory bioactive compounds produces the dual benefit of reducing heightened inflammatory tone and rescuing diminished β2 adrenergic tone, resulting in the improvement of myoblast function and muscle growth in the IUGR fetus.

## 5. Conclusions

From this study, we can conclude that systemic fetal inflammation plays a major role in IUGR muscle growth deficits and, thus, may be an effective target for therapeutic intervention strategies. It is reasonable to assume that placental insufficiency-induced hypoxemia was the primary driver of heightened inflammation in our IUGR fetuses. However, these fetuses also exhibited marked reductions in endogenous circulating eicosapentaenoic acid concentrations. When concentrations of this anti-inflammatory ω-3 PUFA were brought back to normal with daily fetal infusions, indicators of heightened inflammatory tone likewise returned to normal despite the persistence of hypoxemia. After receiving eicosapentaenoic acid infusions for 5 days, IUGR fetuses exhibited a ~50% recovery in muscle growth indicators, which was notably associated with improved myoblast differentiation capacity. Programmed changes identified in IUGR fetal muscle included greater IL6R and reduced β2 adrenoceptor, both of which help explain poor muscle growth. Targeting systemic inflammation with eicosapentaenoic acid failed to resolve the increase in IL6R, which would have reflected circumvention of enhanced muscle sensitivity to inflammation. However, eicosapentaenoic acid unexpectedly recovered the β2 adrenoceptor deficit observed in the IUGR muscle, which likely played a key role in the more favorable growth phenotype. Together, these findings demonstrate the potential value of systemic fetal inflammation as a therapeutic target for improving growth and body composition outcomes in stress-induced IUGR fetuses.

## Figures and Tables

**Table 1:** Body, muscle, and organ masses from heat stress-induced IUGR fetal lambs after 5-day intravenous administration of the ω-3 PUFA eicosapentaenoic acid (EPA).

Group	Experimental Group			
	Control	IUGR	IUGR+EPA	*p*-Value
n	11	8	9	
Absolute Mass, g				
Whole Fetus	3004 ± 130 ^a^	2334 ± 170 ^b^	2650 ± 194 ^ab^	0.01
Hindlimb	298 ± 14 ^a^	222 ± 13 ^b^	262 ± 21 ^a^	<0.01
* Semitendinosus*	6.03 ± 0.44 ^a^	4.61 ± 0.29 ^b^	4.92 ± 0.49 ^ab^	0.03
* Soleus*	1.03 ± 0.13 ^x^	0.63 ± 0.11 ^y^	0.89 ± 0.15 ^z^	0.09
* Longissimus dorsi*	60.5 ± 3.3 ^a^	45.6 ± 3.8 ^b^	50.7 ± 3.8 ^ab^	0.01
* Flexor Digitorum Superficialis*	5.58 ± 0.38 ^a^	3.94 ± 0.46 ^b^	4.58 ± 0.56 ^ab^	0.03
Heart	26.4 ± 1.2 ^a^	22.1 ± 1.4 ^b^	22.0 ± 1.4 ^b^	0.04
Lungs	102.3 ± 4.5 ^a^	78.4 ± 5.4 ^b^	89.9 ± 5.9 ^b^	0.01
Liver	114.7 ± 8.5	99.2 ± 10.2	95.2 ± 9.1	NS
Kidneys	21.1 ± 1.4 ^a^	16.1 ± 1.2 ^b^	18.3 ± 2.5 ^ab^	0.05
Brain	44.6 ± 1.2 ^a^	40.0 ± 1.3 ^b^	41.3 ± 1.7 ^ab^	0.05
Mass/Fetal Mass, g/kg				
Heart	8.8 ± 0.5	9.8 ± 0.6	8.5 ± 0.6	NS
Lungs	33.9 ± 1.2	34.6 ± 1.4	33.6 ± 1.3	NS
Liver	39.1 ± 3.5	46.6 ± 4.1	34.2 ± 3.8	NS
Kidneys	6.8 ± 0.5	7.0 ± 0.9	6.9 ± 0.6	NS
Brain	14.7 ± 0.7 ^a^	16.8 ± 1.1 ^b^	14.4 ± 0.8 ^a^	0.04

^a,b^ Means with different superscripts differ (*p* < 0.05). IUGR, intrauterine growth restriction; NS, not significant. ^x,y,z^ Means with different superscripts tend to differ (*p* < 0.10).

## Data Availability

The datasets generated by this study will be made available upon reasonable request to the corresponding author. The data are not publicly available due to privacy.

## Supplementary Materials

The following supporting information can be downloaded at https://www.mdpi.com/article/10.3390/metabo14060340/s1, Supplemental Figure S1. Hematology components for IUGR fetal lambs administered daily with eicosapentaenoic acid. Supplemental Figure S2. Representative images for myoblast staining in IUGR fetal lambs administered daily with eicosapentaenoic acid.
