# Supplementary material for: Daily Eicosapentaenoic Acid Infusion in IUGR Fetal Lambs Reduced Systemic Inflammation, Increased Muscle ADRβ2 Content, and Improved Myoblast Function and Muscle Growth
Source: Metabolites. 2024 Jun 18;14(6):340. doi: 10.3390/metabo14060340 (PMC11205652; doi:10.3390/metabo14060340)
Supplement: Supplementary file 1 [file metabolites-14-00340-s001.zip › metabolites-3033492-supplementary.pdf]

Supplemental Materials

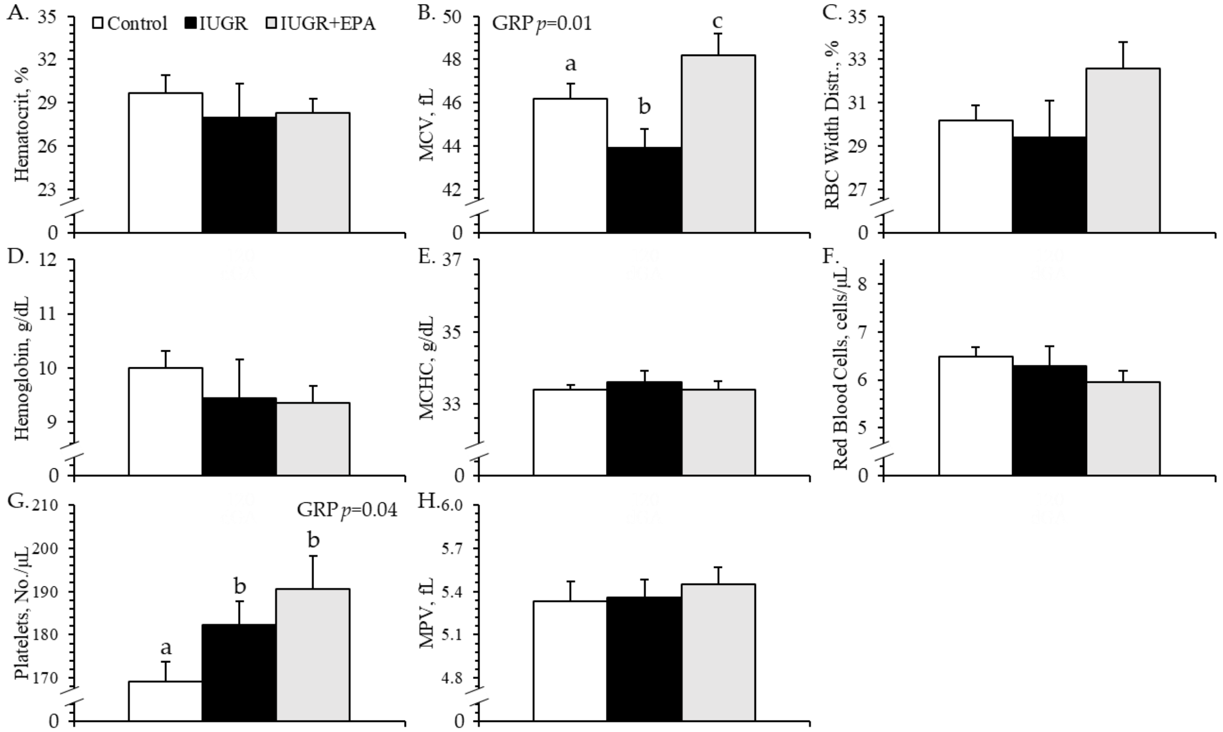

Supplemental Figure S1. Hematology components for IUGR fetal lambs administered daily eicosapentaenoic acid. Daily whole blood samples were collected from control (n = 11), IUGR (n = 8), and IUGR+EPA fetuses (n = 9). Data are presented for circulating hematocrit (A.), mean corpuscular volume (B.), red blood cell distribution width (C.), hemoglobin (D.), mean corpuscular hemoglobin concentration (E.), red blood cells (F.), platelets (G.), and mean packed cell volume (H.). Effects of experimental group (GRP), day of gestation, and group x day interaction were evaluated and are noted where significant ($p < 0.05$). [a, b, c] Means with different superscripts differ ($p < 0.05$).

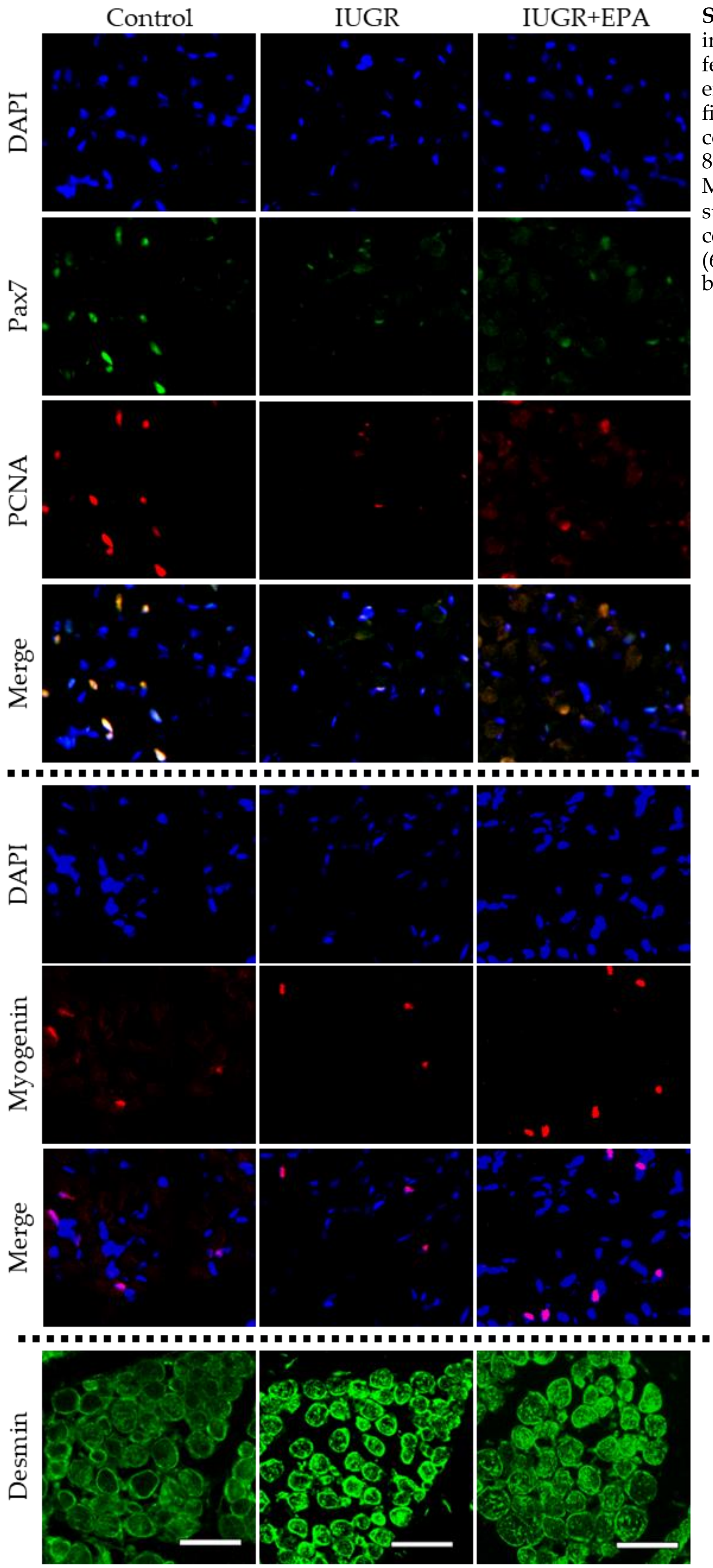

Supplemental Figure S2. Representative images for myoblast staining in IUGR fetal lambs administered daily eicosapentaenoic acid. Cross sections of fixed *semitendinosus* muscles were collected from control (n = 11), IUGR (n = 8), and IUGR+EPA fetuses (n = 9). Micrographs are presented that show staining for pax7 (2nd row), proliferating cell nuclear antigen (3rd row), myogenin (6th row), and desmin (bottom row). Scale bar = 50 µm.
